# Cost-effectiveness decision modelling in social care: exploring the why, the how, and the what next

**DOI:** 10.3310/nihropenres.13789.2

**Published:** 2025-07-17

**Authors:** Dacheng Huo, Sebastian Hinde, Laura Bojke, Annette Bauer, Helen Weatherly

**Affiliations:** 1 University of York Centre for Health Economics, York, England, UK; 2 The London School of Economic and Political Science Care Policy and Evaluation Centre, London, England, UK

**Keywords:** Social care, social care data, economic evidence, decision modelling

## Abstract

**Context:**

In social care research economic evaluation has had limited impact, in contrast to other health related areas. However, increasing research funding and policy interest is occurring, including regarding the role of cost-effectiveness decision modelling.

**Objectives:**

We explore why cost-effectiveness decision modelling is informative in a social care setting, how it can and has previously been implemented, and what next steps are needed to ensure consistent, robust, and informative models are produced to inform social care decisions.

**Method:**

This paper consists of an overview of the theoretical added value of cost-effectiveness decision modelling in a social care setting, alongside a literature search summarising the key features of decision models in the current published and grey literature.

**Findings:**

Cost-effectiveness decision modelling in social care is relatively undeveloped with only a few examples identified and minimal methodological research in the area. These studies varied greatly in the approaches taken but demonstrate the practicality and value of decision modelling.

**Limitations:**

The pragmatic approach to the literature review may have missed some existing decision models but we consider the findings to be appropriate.

**Implications:**

Cost-effectiveness decision modelling has the potential to play an important role in informing effective, consistent, and transparent decision-making processes in social care. However, methodological developments are needed to standardise the approaches taken.

## Plain Language Summary

This article focuses on a method called "cost-effectiveness decision modelling," which can help improve decision-making in social care. Social care provides essential services to people who need help with daily activities due to age, illness, or disability. Even though economic evaluation is common in other areas of health, it hasn’t been widely used in social care, where the need for thoughtful use of limited resources is just as important. Recently, however, there’s been more interest in using cost-effectiveness models to inform decisions about which services provide the best value for the resources spent.

In this article, we explore why decision modelling is important for social care, how it has been used in the past, and what needs to happen to make it a more reliable and useful tool. Decision modelling brings together information from different sources, like research studies and expert opinions, to help predict the costs and benefits of different services. By comparing different care options, decision modelling can show which services deliver the best outcomes for individuals and the community.

Our review of existing research found that decision modelling in social care is still quite limited, with only a small number of examples available. The studies we found used different approaches, which makes it difficult to compare their results. However, these models show the potential of decision modelling to improve how resources are allocated in social care.

To make this tool more effective, further research is needed to standardize the methods used and ensure that models are robust and reliable. With these improvements, decision modelling could play an important role in helping policymakers and practitioners make better, more informed decisions about social care services.

## Introduction

Social care, also known as ‘long term care’ outside of the UK, provides social work, personal care, protection and social support services to children and adults who are deemed to be in need or at risk due to illness, disability, frailty, or poverty. As well as supporting service users or clients, it can support informal unpaid carers, such as family members of the service user. In the UK, social care receives funding through the statutory (public) sector, however supply is limited and need for care is routinely means tested, resulting in many users self-funding. The voluntary, community, and social enterprise as well as the private sector are important partners for statutory health and social care agencies in providing social care, meaning that public, private, and third sectors are all part of the delivery of social care.

The objectives of adult social care in England, and responsibilities of local authorities in relation to social care, are set out in the Health and Social Care Act ( Glover-Thomas, 2013). Key objectives of social care include supporting individuals to remain independent in the community, supporting their dignity, personal choice, and control over their daily lives, as well as supporting social-care related quality of life and safety. More broadly, social care supports the health care system with its role in prevention and reducing health care service use. Alternative outcome measures relevant to social care, such as the Adult Social Care Outcomes Toolkit (ASCOT), ICEpop CAPability measure of older people ICECAP-O, and ICECAP-A (for adults), provide valuable metrics for capturing the broader impacts of social care interventions beyond traditional health outcomes.

Despite this potential and the well-established use of economic evaluation in health care, we believe its application within social care research and practice has been limited. The primary purpose of this paper is to identify further research needs in the field of economic evaluation within social care and to provide comprehensive guidance for researchers and practitioners interested in the methodologies of social care interventions. This paper highlights the potential value of modelling in the evaluation of social care service. Through a purposeful review of existing modelling studies, we aim to offer a clearer understanding of the current landscape, methodological strengths and gaps, and encourage the development and application of more rigorous, transparent, and context-appropriate modelling approaches in social care settings.

## Challenges of cost-effectiveness analysis in social care

Cost-effectiveness analysis, the summation of the costs and effects of alternative courses of action, is considered an informative component in the development of National Institute for Health and Care Excellence’s (NICE) guidance related to best social care provision in England ( NICE, 2014). By seeking to estimate the value gained from resources used to implement an intervention or policy, cost-effectiveness analysis aims to determine the best alternative use of limited resources, and the benefits that would have been gained from taking the alternative course of action. 

The challenges of conducting robust cost-effectiveness analysis in social care, in contrast to the other areas such as health technology assessment, have previously been defined and discussed ( Bauer *et al.*, 2021; El-Banna *et al.*, 2021; Suh & Holmes, 2022; Weatherly *et al.*, 2017). The challenges include: defining the objectives of the intervention and services, and relatedly, the complexity of outcomes of interest, the range of stakeholders in the public, private, and third sectors, complexity of interventions, and availability of research and data. These challenges are especially evident when compared to the evaluation of new pharmaceuticals, for which the methodological approaches to conduct such evaluations were originally developed and for which the consideration of health maximisation subject to costs borne by the public healthcare system are considered sufficient ( Drummond *et al.*, 2015). Furthermore, reviews of cost-effectiveness analyses of social care interventions ( Bauer *et al.*, 2021; Weatherly *et al.*, 2017) reveal that an increasing number are being undertaken in the social care field but with heterogeneity in methodological approaches used, raising the question of limited standardisation of evaluative methods.

Establishing the cost-effectiveness of an intervention, broadly employs one of two approaches with which to generate findings: empirical data analysis from a single study and decision modelling ( Drummond *et al.*, 2015). The use of empirical data relies on evidence generated from sources such as clinical trials and observational and routinely collected data. In contrast, in decision modelling the pathways of care are simulated, and multiple data sources used to impute costs and benefits as they are assumed to occur at different decision-making points or key events ( Briggs *et al.*, 2006). This method allows extrapolating costs and effects beyond the time-period of an experimental study.

This paper explores *why* cost-effectiveness decision modelling has the potential to be informative in a social care setting, *how* it can and has previously been implemented, and *what next* steps are needed to ensure consistent, robust, and informative models are produced to inform social care decisions. To do so, first we introduce decision modelling as an approach, exploring its distinctive features and aims and its conceptual value to inform decision making in social care. Through a purposeful review of existing published models, we consider how decision modelling has been operationalised in social care to date and finally seek to define a route forward to increase the usefulness and application of decision modelling in social care.

## *Why* cost-effectiveness decision modelling has a role in social care

To evaluate the effectiveness and cost-effectiveness of a commissioning decision it is necessary to understand what the aims and objectives are of all relevant stakeholders and consider how the cost and outcomes of interest to each are impacted by different available commissioning decisions ( Drummond *et al.*, 2015) Given the potential for social care interventions to have multiple decision makers, sectors, objectives, and budgets, this can be challenging. Decision modelling can address many of the conceptual and logistical challenges of conducting cost-effectiveness analyses of complex, real-world interventions. Decision, or ‘economic’ modelling, is a broad term to describe the use of mathematical analysis to simplify the complexity of real-world situations. In health economics decision modelling has been defined as the use of such analyses to estimate a series of possible health outcomes and resource use implications which would result from alternative interventions ( Briggs *et al.*, 2006).

In practice, decision models are created by conceptualising and simplifying a complex reality in which interventions exist, focussing on the areas where a demonstrable difference exists between the alternative course of action. This simplification focuses on a limited number of definable states in which the individual can exist. These may be health states, e.g. pre-frail, frail, and dead, or service states, e.g. living at home, admitted to care home, admitted to hospital. Movement between these states, and the impact of each mode of care being evaluated on these transitions is then estimated, with the outcomes of interest and resource use implications of each state reported. The model can then be ‘run’ over the time-period considered relevant, and the sum of the outcomes and resource use implications estimated and compared.

The approach is commonly implemented to inform the deliberations of a national commissioner of health services where the quality adjusted life years (QALYs) of the treated individual are the primary outcome of interest ( Briggs *et al.*, 2006). While a healthcare QALY based model may not be of primary interest in social care commissioning deliberations, a social care QALY based model might do ( Stuttard *et al.*, 2021). For instance, employing an outcome instrument like the ASCOT, which better reflects social care outcomes, permits assessment based on impacts to social care-related quality of life. This means evaluating services on improvements to independence, social connection, or control, rather than just clinical health metrics. Decision modelling as an approach is flexible so long as it is possible to conceptualise and estimate the relevant states individuals may occupy, the costs and outcomes associated with their membership, how they transition between them, and the impact of competing modes of care.

Decision modelling approaches can be argued to have five key benefits in informing deliberations in any setting ( Harris *et al*., 2015):

1) Bringing information together from multiple sources
2) Extrapolating over the longer term, and more specifically into the future
3) Making comparisons across relevant interventions – making more efficient use of data
4) Characterising and evidencing uncertainty
5) Informing multiple stakeholder perspectives

In this section we explore the role of each of these elements related to social care decision making.

### Bringing information together from multiple sources

A substantial challenge of conducting cost-effectiveness analysis in social care is the limited availability of evidence which can be considered robust in isolation, although this is improving ( Tinelli *et al.*, 2020) Moreover, in social care, evidence is often fragmented across different agencies and sectors, with data collected independently and for varying purposes. Decision models allow the integration of these disparate sources, including qualitative data, expert judgment, and observational studies, into a unified evaluative framework. This is particularly crucial in cases where traditional health-related metrics (e.g., QALYs) are insufficient to capture the full breadth of social care outcomes. However, decision modelling provides a framework to both synthesise evidence from a disparate range of sources but also to test the sensitivity of the conclusion to changes in the informative data.

Such data may include trial data but could also include routinely collected data, expert opinion, or exploratory scenario analysis where little is currently known about the value of a parameter. Data suitable to inform cost-effectiveness analysis are currently less common in social care, with evidence on best practice having developed in unstructured ways that are experience-based rather than experiment-based ( Gould & Kendall, 2007; Shields & Elvidge, 2020; Suh & Holmes, 2022). Furthermore, some have argued that as a field of research social care is not sufficiently developed to meaningfully develop what could be considered unbiased estimates through approaches such as RCTs, necessitating the use of alternative sources of evidence ( Rosten, 2020). However, the social care economic evidence base is expanding ( Tinelli *et al*., 2020) as seen in the ESSENCE compendium (described below). Additionally, more administrative data is available, and methods such as expert elicitation have been developed to inform the necessary structures and parameters of decision models which can be applied to a social care setting ( Bojke *et al*., 2021).

### Extrapolating over the longer term, and more specifically into the future

Many of the reasons for providing a service, be it social care or health care, are to improve longer-term outcomes. This may be to strengthen an individual’s activities of daily living over the next month, or to improve their chance of being robust to frailty in a decade’s time. Decision modelling allows this bridge between intervention today and outcomes over the longer-term to be made explicit, and for any necessary conditions for outcomes to be achieved, made clear ( Knight *et al*., 2016). While in social care many of the interventions are designed to deal with immediate care needs, such as personal care, areas of intervention such as home adaptation and reablement services entail intervention now, to support living independently within the community and to improve potential outcomes in the future. While their aim may be to reduce the risk of catastrophic deterioration (requiring hospitalisation or institutional care) rather than any curative intent as is often applied in a health care setting, consideration of effect beyond the initial intervention period represents an important element in considering the value of a service. Social care can also delay access to more intensive services such as residential homes and may facilitate earlier discharge from hospital.

### Making comparisons across relevant interventions

Cost-effectiveness analyses which only include a limited set of the full range of competing modes of care risk drawing inefficient or erroneous conclusions by potentially recommending one option as cost-effective when better options may be available but were excluded from the analysis ( NICE, 2013) for example in a two-armed trial which omits other relevant comparators. Decision modelling provides a framework to overcome these challenges through its ability to draw from multiple sources of evidence directly or use methodological approaches such as scenario analysis and meta-analysis.

While not unique to the area, one of the challenges of social care is that it represents a complex set of modes of care, especially compared to pharmaceutical interventions which can easily be characterised into the medication and doses provided. Relevant to this, the NICE Social Care Guidance ( NICE, 2013) recommends that all modes of care ‘routinely delivered by the public and non-public social care sector’ should be incorporated in any economic evaluation. Coupled with the challenges of conducting trial analysis in this setting such a requirement typically would necessitate the use of decision modelling.

### Characterising uncertainty

An important strength of cost-effectiveness analysis is its ability to inform decision makers about the level of uncertainty in conclusions drawn. Decision modelling facilitates this through probabilistic and deterministic sensitivity analyses. These approaches draw on information beyond the expected average costs and benefits of the relevant interventions to demonstrate the uncertainty around the conclusion of the evaluation. This uncertainty can be expressed in several ways including the probability of the decision being incorrect, the impact of such a wrong decision, and whether the cost and delay of additional research can be justified to reduce our uncertainty. Evidence to inform such analyses can be drawn from the primary source, for example a trial, wider evidence, or exploratory scenarios. The comparatively limited level of research evidence and level of complexity regarding causal pathways of care evident in social care implies the consideration of uncertainty is of importance.

### Inform multiple stakeholder perspectives

To be useful in the decision-making process, cost-effectiveness analysis must reflect the needs of the key stakeholders ( Williams & Calnan, 1991). However, defining who the key stakeholders of an evaluation are in social care can be difficult, potentially spanning all sectors of the economy. Social care-related services are delivered by a range of providers, including various public sector agencies, commercial providers, the voluntary and community sector, and unpaid carers. Furthermore, services may be provided in-house or externally contracted, for example with the commissioning of vision rehabilitation services by local authorities ( Longo *et al.*, 2020). The costs are also borne by various public sector agencies, service users and their families. As a result, the payer-provider matrix is complex and differs across the range of social care services available; some social care is funded by the local authority, others by service users or a mixture of both, and some social care is provided by carers or volunteers. This is further complicated by often strong correlations between health and social care activities, for example the lack of social care provision may delay discharge of individuals from hospitals.

By explicitly conceptualising the movement of an individual through a finite number of states and estimating the payoffs as these transitions occur, decision modelling allows the analysis to be re-run from different perspectives. For example, costs borne from a health care perspective can be recorded separately from a social care or a private individual payer’s perspective.

## How has decision modelling previously been implemented in social care

In England, since 1999, NICE has provided national guidance on the promotion of good health and the prevention and treatment of ill health. Under the Health and Social Care Act 2012 NICE was given additional responsibility to develop guidance and quality standards for social care, making it the first health technology agency internationally to do so. As part of its remit NICE developed a reference case for the economic evaluation of interventions with a social care focus, a generalisable specification of the methods considered appropriate ( NICE, 2014). The reference case has been summarised elsewhere, a key finding of which is that compared to HTA guidance, economic evaluation methods guidance for use in social care is much less prescriptive and the range of suggested methods much broader ( Bauer *et al.*, 2021; Weatherly *et al.*, 2017). Published systematic reviews have shown that over recent years more economic evaluations of social care interventions have been undertaken, but that the scale is still limited ( Tinelli *et al.*, 2020; Weatherly *et al.*, 2017).

The latest general NICE reference case provides some guidance regarding the appropriate approach to conducting decision modelling to inform cost effectiveness analysis ( NICE, 2014) and there are existing good practice guides when conducting decision modelling informed cost-effectiveness analysis which have focussed on health technology assessment ( Briggs *et al*., 2006). However, the extent to which these are generalisable to social care of implemented is unclear.

On the other hand, although several reviews have examined economic evaluations in the field of social care, they tend to concentrate on cost-effectiveness results within specific population groups or service areas, without providing a systematic analysis of the modelling methodologies employed. For example, Suh and Holmes (2022) critically reviewed existing cost-effectiveness research in children's social care, highlighting key methodological and policy challenges. El-Banna *et al*. (2021) conducted a systematic review of economic evaluations of children’s social care interventions, synthesising evidence across a range of programme types. Rizzo and Rowe (2016) focused on social work services for ageing populations, providing an updated review of their cost-effectiveness. As a result, there remains a lack of clarity regarding how modelling is conducted in the broader context of social care.

## A compendium of economic evidence in social care

Economics of Social Care Compendium (ESSENCE) Toolkit, funded by the NIHR School for Social Care Research (SSCR), is a comprehensive repository of economic evaluations and decision models in the adult social care sector ( Tinelli *et al*., 2020). The project is designed to support evidence-based decision-making within England's social care system. It systematically gathered and built economic evidence that is relevant to the social care practice in England, with a strong emphasis on economic evaluations. The economic evidence included in the ESSENCE Toolkit was drawn from relevant databases identified through an iterative selection process guided by the project’s advisory group. A detailed account of the methodology—covering the glossary of terms and concepts employed, data sources, composition of the expert team, and criteria for assessing the type and robustness of the included evidence—is provided in Tinelli *et al*. (2020).

A key strength of the ESSENCE Toolkit lies in its broad definition of adult social care, which is taken from a framework developed by National Audit Office ( NAO, 2018). It includes economic evidence on a diverse set of interventions across various service user groups, settings, and caregiver contexts. Although the toolkit’s primary focus was adult social care, now it includes economic evidence on children's social care interventions too. Another advantage of the ESSENCE Toolkit is that it goes beyond simply summarizing existing evidence. It addresses gaps in the literature by generating new findings through modelling techniques, providing insights where evidence is lacking.

## Purposeful review method and findings

To explore the scale and scope of decision modelling studies in social care, we conducted a purposeful review of published studies listed in the ESSENCE Toolkit. It provided a readily available evidence base, allowing for a targeted and efficient review aligned with our research objectives, in contrast to a broader, less focused search with uncertain additional value.

The literature selected in this study are those that met the following criteria:

1) Conducted economic evaluations of social care interventions, using the NHS England definition of social care as practical support provided because of illness or disability ( NHS, 2021). The broader term of ‘economic evaluation’ was used to ensure any relevant models which conducted similar analyses such as cost-benefit or cost-consequence were also included; and
2) employed decision modelling methods such as decision trees, Markov decision processes, or simulated modelling to estimate the performance of the programme.

We are aware that there is also a literature describing the use of agent-based modelling in social care to, for example, predict social care demand ( Gostoli & Silverman, 2019; Gostoli & Silverman, 2022) however these do not extend to cost-effectiveness analyses, nor do they evaluate specific interventions and thus for brevity were excluded from this review. However, they are informative when considering available methodological approaches to decision modelling in this setting. Future research should explore integrating agent-based models to capture the multifaceted nature of social care interventions.

We conducted a quality assessment of the included studies using the Philip’s checklist criteria to evaluate the robustness of their decision modelling approaches. Despite the purposeful nature of this review, incorporating a quality assessment helps mitigate potential biases and provides a more comprehensive understanding of the existing literature, covering aspects such as model structure, data inputs, consistency checks, and uncertainty analysis ( Philips *et al*., 2004). The reason the Philips' checklist was chosen over the Drummond checklist is that it provides a more specialized focus on the modelling evaluations ( Min *et al*., 2021).

This approach identified 55 studies from an initial search of the titles by criteria 1), with eight being considered relevant to the aims of this paper ( Bauer *et al.*, 2010; Bauer *et al.*, 2017; Dixon *et al.*, 2014; Mavranezouli *et al.*, 2014; McDaid *et al.*, 2017; Public Health England, 2018; Tong *et al.*, 2017), one of which contained six independent models ( McDaid *et al.*, 2017). The purposeful nature of this review implied pragmatic inclusion and exclusion criteria but broadly the aim was to identify economic evaluations of social care interventions which incorporated some element of a clearly described decision model. We extracted data on key aspects such as the type of decision model used, perspective adopted, time horizon, data sources, measures of effect, uncertainty analysis, headline results, and identified challenges. This systematic extraction process ensured consistency and comprehensiveness in capturing relevant information from each study. The majority of the 55 studies identified as economic evaluations did not incorporate a decision modelling element, consisting of estimates of cost and benefits from primary data sources, for example trials. While not a complete description of the published decision models in social care we consider these eight studies to be an indication of the state of play in the discipline sufficient for the aims of this paper. A summary of the models produced is available in Supplementary Table 1 (Refer extended data), and the result of the quality check is presented in Supplementary Table 2 (Refer extended data).

There is a wide range of approaches taken to modelling in social care, despite the relatively small number of available studies (see Supplementary Table 1). Approaches range from relatively simple decision trees exploring outcomes over a short time horizon ( Bauer *et al*., 2010) to individual/client level simulations over the lifetime of the individual ( Tong *et al.*, 2017). In general, many of the decision models considered a short time horizon, for example a year, focussing on the period of intervention, with most limiting their horizon to five years or less. This was consistent with the model design and nature of the intervention being evaluated, with the case made that the costs and benefits would only occur over the short term, e.g. support at home services ( Dixon *et al.*, 2014) and falls prevention ( Public Health England, 2018).

The models included evidence from a wide range of sources, primarily the published literature but also evidence from trials ( Cottrell *et al.*, 2018; Mavranezouli *et al.*, 2014), observational data ( Dixon *et al.*, 2014), and hypothetical values ( Dixon *et al.*, 2014) were used. Most model parameters were drawn from published literature or relevant trial data. While the more complex models reviewed included extensive uncertainty analysis, including probabilistic and scenario-based sensitivity analyses ( Bauer *et al.*, 2017; Cottrell *et al.*, 2018; Tong *et al.*, 2017) the majority of the models focussed on a single base-case analysis. Where it was presented, uncertainty was only explored in terms of impact of the headline results of the analysis with no exploration of advanced modelling approaches such as value of information methodologies or distributional cost-effectiveness analysis, methods which seek to estimate the value of investing in additional research and the differential impact of interventions by socio-economic factors respectively ( Weatherly *et al*., 2017).

As the Philips checklist shows, most evaluations clearly state perspectives, model structures, and justify data inputs. However, common limitations persist, particularly in addressing uncertainty, heterogeneity, and extrapolation methods. Few studies justify the exclusion of options or incorporate alternative structural assumptions, highlighting the need for more transparent handling of uncertainty and broader methodological rigour in social care economic modelling.

Overall, the published decision models demonstrate the lack of availability of a single appropriate reference case to apply when deciding the optimal decision modelling approach, with a wide range of approaches taken to conduct economic evaluation but importantly no clear logic as to why the different approaches were selected. While reference was made to the complexity of social care decision making in some of the studies (e.g. McDaid *et al.* (2017) and Public Health England (2018)), few attempts were made to reflect this complexity in the decision model, with the focus most often on costs falling on the public sector, specifically NHS and personal social services, and benefits to the intervention recipients. In some cases, a broader perspective was additionally defined as a secondary analysis ( Tong *et al.*, 2017), however, the lack of any widely agreed approach to incorporating the wider implications of social care beyond the public sector cost and health implications makes the extension of any decision model challenging. However, incorporating broader economic evaluation methods such as cost-benefit analysis or cost-consequence analysis could provide a more comprehensive understanding of the multifaceted impacts of social care interventions.

## Discussion: what is next for decision modelling in social care?

Economic evaluation is increasingly being applied in social care settings to provide valuable insights to inform the effective and cost-effective use of limited resources. Decision modelling represents a valuable tool to synthesise and analyse data for economic evaluation as it facilitates the incorporation of evidence from multiple sources, the extrapolation of evidence over a longer period than may be directly available, comparisons across all relevant interventions, and the characterisation of uncertainty.

Modelling has been employed in social care programs with examples of highly complex models ( Tong *et al.*, 2017) and attempts to incorporate the complex set of stakeholders and relevant outcomes evident with social care delivery in England ( McDaid *et al.*, 2017). However, as demonstrated by our review, the overall scale of such models is limited, with only eight separate studies being identified as having implemented decision models relevant to a social care setting. There are many potential reasons for this lack of scale, but key challenges facing decision modelling in this setting can be considered in three elements: contextual, methodological, and evidential ( Squires *et al.*, 2016).

In addition to the challenges facing economic evaluation of social care more broadly, and discussed elsewhere ( Bauer *et al.*, 2021; Weatherly *et al.*, 2017), the contextualisation of the decision problem and care pathway represents a key challenge for decision modelling in this area. The wide variation in the form of intervention and real-world service provision makes summarising the decision problem in a robust way in the form of a decision model much more challenging in social care, with the risk being the issue is over simplified.

While a key strength of decision modelling techniques is the ability to draw information from a range of sources including trial, observational, and expert elicited data, the complexity of the evidence requirement and relatively limited history of data collection in social care settings, limits the opportunity to conduct meaningful decision modelling in this setting. Efforts are, however, being made to better report and make available routine data in social care (e.g. The Catalogue of Social Care Data ( CPEC, 2023)) and to increase the level of funding of research for example by NIHR in the UK ( NIHR, 2022).

In recent years there has been significant progress made in methodological developments related to conducting economic evaluation in more complex areas of health and social care provision, for example Skivington *et al.* (2021) provide a framework for developing and evaluating complex interventions and economic considerations are considered core in determining the comparative resource and outcome consequences of the interventions for those people and organisations affected. They suggest that broad economic approaches to evaluation such as cost-consequence analysis or cost-benefit analysis might be relevant to capture the full range of non-health as well as health costs and benefits across different sectors, and NICE ( 2013) ( 2014) also supports use of these economic evaluation approaches of complex intervention. Squires *et al.* (2016) have produced a similar framework when conducting decision modelling applied to public health. However, there are still numerous areas still requiring methodological development including the modelling of impacts across different sectors and spillover effects (e.g. accounting for the network of support including family and friends), the incorporation of social care specific outcome measures into decision modelling, and the relevant opportunity cost threshold in social care.

While these are all issues that have contributed to the limited application of decision modelling in social care it is evident that it is not the fundamental nature of decision modelling to which they apply, rather to the broader challenges of conducting robust evaluations in a social care setting. As the models identified in the pragmatic review demonstrate, the use of decision modelling in social care is both possible and informative to the setting. Modelling is well used in these studies to highlight the potential costs and benefits of interventions and importantly identify where data is lacking ( Dixon *et al.*, 2014).

In terms of ‘ *what next for decision modelling in social care’,* our review has demonstrated that a range of economic evaluation methods and modelling approaches are being used, applied to social care. The range of methods used is consistent with NICE methods guidance in social care. As a tool for undertaking cost-effectiveness analysis, models offer a flexible and somewhat creative approach to undertake rigorous analysis, using available data to best serve the needs of the relevant stakeholders. However, while the current flexibility in approaches has ensured relevance of each decision model to the setting in which it is applied, care is needed that this is not to the detriment of comparison across the findings of each model. Furthermore, understanding how local authorities interpret the results of cost-effectiveness analyses is crucial. The national versus local perspective disconnect, articulated by Hinde *et al.* (2020). Local authorities often focus on cost savings and resource allocation, which may influence the prioritization of interventions. National CEA frameworks prioritising long-term QALY maximisation using fixed discount rates and specific thresholds often fail to address local concerns about short-term budgets, affordability risk, and broader social objectives like equity ( Hinde, 2024). This necessitates specific adaptations in how models are built and reported for local audiences (e.g., scenario analyses on time horizons and explicit budget impact reporting) and requires training to focus on assessing the local applicability of any economic evidence presented. Providing clear guidelines on interpreting CEA results can also help mitigate potential misinterpretations and ensure informed decision-making.

In the context of social care, relying solely on conventional outcome measures such as QALYs may not fully capture the primary benefits of many interventions. As such, alternative outcome measures with a better conceptual fit should be considered. Measures such as the ASCOT, SCRQoL and ICECAP, which reflects broader notions of capability wellbeing, may offer more appropriate frameworks for evaluating the outcomes of social care interventions ( Stuttard *et al.*, 2021).

Another area ripe for future development is the inclusion of equity and distributional considerations within decision models. Current approaches largely focus on average outcomes, but there is increasing recognition that social care interventions often benefit different population subgroups in varying ways. Incorporating distributional cost-effectiveness analysis into future models would allow for more nuanced conclusions about how benefits and costs are distributed across different socio-economic groups, ensuring that decision-makers can account for equity when prioritising resources.

Some hybrid modelling approaches that have been widely used in the cost-effectiveness analysis of other areas such as vaccines and infectious diseases may also be useful for evaluating social care interventions. For example, although the studies by Gostoli and Silverman ( 2019; 2022) did not carry out a cost-effectiveness analysis, their use of ABM to predict social care demand shows that these models can be applied in this area. It might also be worthy to explore the value of some integrated models which incorporate the strength of other models such as discrete event simulation (DES), system dynamics (SD), and ABM etc. These models are helpful for evaluating complex interventions, especially those involving different groups of people and complicated service processes. Using such models in social care can make economic evaluations more realistic and useful for decision-makers.

Social care systems are characterised by long-term trajectories, complex interactions, high heterogeneity, and intricate systems. Although this purposeful review didn’t capture the hybrid models value of alternative modelling methods may also be explored such as microsimulation models, discrete event simulation (DES), system dynamics (SD), and ABM. Microsimulation captures individual-level heterogeneity and long-term outcomes; DES focuses on resource flows and service processes; SD highlights systemic feedback loops and delayed effects; and ABM models micro-level behavioural interactions that shape macro-level system patterns. These models allow for a more nuanced simulation of interventions, particularly those with high heterogeneity or involve complex service delivery pathways.

## Limitations

This review adopted a pragmatic approach, which may have resulted in some relevant decision models being overlooked. However, we believe the findings are still appropriate, as the ESSENCE Toolkit provided a strong foundation for evidence gathering. Another limitation is related to the definition of social care used in this study. Due to the lack of a consistent definition in the literature, we followed the broad definition adopted by ESSENCE. While this allowed us to include a wide range of relevant studies and modelling techniques, it also means that some of the interventions reviewed may not fall within the narrower, working definition of social care—typically limited to services commissioned or delivered by local authority social services departments. Although there may be some overlap with other sectors, this broader scope also provides a useful overview of the current landscape of modelling in social care.

## Conclusions

George Box’s eponymous statement that ‘all models are wrong; some are useful’ could be argued to never have been as fitting as in the setting of decision modelling in social care, a setting in which complexity and immaturity of data and research combine to limit the ability to create robust decision models. However, as has been shown through the models that do exist in this setting, there is simultaneously significant potential for decision modelling approaches to provide clarity to evaluations in this setting. As increasing research funding ( NIHR, 2022), policy interest, and routine data collection develops, decision modelling will represent an important approach to undertaking economic evaluation in the field.

## Data Availability

No data associated with this article. Figshare: Cost-effectiveness decision modelling in social care: exploring the why, the how, and the what next, DOI: 10.6084/m9.figshare.27612558 The project contains the following extended data: Literature Table - Summary of existing social care decision models Quality Assessment – Philip Checklist Results Data are available under the terms of the Creative Commons Attribution 4.0 International license (CC-BY 4.0).
