# Supplementary material for: Cost-effectiveness decision modelling in social care: exploring the why, the how, and the what next
Source: NIHR Open Res. 2025 Oct 23;5:9. Originally published 2025 Jan 27. [Version 3] doi: 10.3310/nihropenres.13789.3 (PMC12790600; doi:10.3310/nihropenres.13789.3)
Supplement: Peer review history

# Reviewer report: Reviewer response for version 3

**DOI:** 10.3310/nihropenres.15380.r39041

**Published:** 2026-01-10

**Authors:** Jill Manthorpe

**Affiliations:** 1 King’s College London, London, UK

Thank you for the opportunity to read this interesting paper. It makes a strong case for increased and more impactful economic evaluation of social care services and options. The summary of studies will be helpful to others, and the supplementary material useful in summarising the current evidence. There are few comments to make, but perhaps the authors might consider their language so that it chimes with social care perspectives of inclusion and thus makes their arguments more meaningful. For example, people are not 'admitted' to care homes and references to this as being 'institutional care' undermine efforts to be person-centred and the exercise of choice. For economists, residential care may be 'intensive' but this is not generally experienced as such, expensive maybe ...

 There are a few remaining typos in the text eg in the paragraph starting 'The latest general NICE ...' the end of this paragraph would seem to need OR not OF. Programmes are generally used in UK social care not programs. The term 'personal social services' is very dated now and only used in rarefied documents or data. Elsewhere social care is used appropriately, so if PPS is being used to mean LA funded services that could be said, not forgetting of course that many people pay for these or have state benefits reduced if in receipt.   

 The discussion centres round three helpful themes. There might be a case for economists to be more social care 'aware' through professional development and early education/training. This may be encouraged by the investment in social care research but earlier equipping of economists with social care awareness might be helpful and of course more attention in social care skills development to economics would likely be mutually helpful.

Is the review written in accessible language?

Yes

Are all factual statements correct and adequately supported by citations?

Yes

Are the conclusions drawn appropriate in the context of the current research literature?

Yes

Is the topic of the review discussed comprehensively in the context of the current literature?

Yes

Reviewer Expertise:

social care

I confirm that I have read this submission and believe that I have an appropriate level of expertise to confirm that it is of an acceptable scientific standard.

# Reviewer report: Reviewer response for version 2

**DOI:** 10.3310/nihropenres.15245.r36694

**Published:** 2025-09-08

**Authors:** Willem Kuyken

**Affiliations:** 1 University of Oxford, Oxford, England, UK

This report reviews how cost-effectiveness decision modelling could improve decision-making in social care, where resources are scarce and this approach is rare. Only a small number of social care studies have used such models, and they vary so much in method that results can’t easily be compared, though they do show the approach’s potential value. The authors argue that standardized methods and stronger evidence are needed to make decision modelling a reliable tool for guiding policy and practice in social care. 

 I found the "evidence is limited, more research needed" rather disappointing. The exploration of why social care seems to use evidence-based approaches much less also seemed a glaring omission, as without that deeper exploration a conclusion "more research is needed" is very unlikely to have any impact. Given NIHR's brief to use research impact health and social care this seems a missed opportunity. While the review is robust, the discussion could do more to explore promising frameworks, approaches that are likely to yield benefits in social care, nothing of course that this needs further research.

 Obvious low hanging fruit are: 

 Smarter allocation of scarce resources – Decision modelling can reveal which services (like reablement, falls prevention, or home adaptations) give the best long-term outcomes for the money. That means fewer wasted resources and more people receiving interventions that keep them independent longer.

 Fairness and equity – Newer modelling approaches (e.g., distributional cost-effectiveness analysis) can show how benefits are spread across different groups (e.g., by age, disability, or income). This allows decision-makers to prioritize not just efficiency but also fairness in service provision.

 The plain English summary could be much improved if a good PPI group were involved. I used OpenAI  5.0 and it generated this:

 "This article looks at a way of making choices in social care called “cost-effectiveness decision modelling.” Social care is about helping people who need support with daily life because of age, illness, or disability. Money and resources are limited, so it’s important to spend them wisely. While this kind of analysis is already common in healthcare, it hasn’t been used much in social care until recently.

 The article explains:

- Why this method matters: It can help decide which services give the best results for the money spent.
- How it works: It combines information from studies, expert opinions, and other sources to predict the costs and benefits of different services.
- What it can show: By comparing options, it can highlight which services give the best outcomes for both individuals and the wider community.

The authors found that only a few studies have used this method in social care so far, and they often use different approaches. This makes it hard to compare results. But the existing examples suggest the method could be very useful. For example, smarter allocation of limited resources and attention to fairness and equity are likely to yield benefits.

 To make it more reliable, more research is needed. The methods need to be standardised (so everyone uses the same approach) and tested to make sure they give trustworthy results. If that happens, decision modelling could become an important tool to guide better choices in social care."

Is the review written in accessible language?

Partly

Are all factual statements correct and adequately supported by citations?

Yes

Are the conclusions drawn appropriate in the context of the current research literature?

Partly

Is the topic of the review discussed comprehensively in the context of the current literature?

Yes

Reviewer Expertise:

Translational mental health research and impact

I confirm that I have read this submission and believe that I have an appropriate level of expertise to state that I do not consider it to be of an acceptable scientific standard, for reasons outlined above.

# Response

**Published:** 2025-10-16

**Authors:** Dacheng Huo

**Affiliations:** University of York Centre for Health Economics, York, England, UK

Dear Reviewer,

 Thank you very much for your thoughtful and constructive comments. We found your feedback extremely valuable in strengthening both the argument and the practical relevance of our paper. Please see our responses below:

**On why social care seems to use evidence-based approaches much less**

 We fully agree that our initial manuscript did not adequately explore this. We have now expanded the introduction to discuss several reasons: the fragmentation of funding and provision across public, private, and voluntary sectors; the broader outcomes of interest (e.g., independence, dignity, wellbeing) which are less amenable to standardised measurement; limited availability of longitudinal or routine data; and the differences between national policy goals and local authority budget priorities. We also highlight how these contextual barriers have shaped the relative lack of decision modelling in social care to date.

**On exploring promising frameworks and approaches**

 We agree this is a valuable direction. In the revised Discussion we now go beyond “more research needed” and identify concrete ways in which modelling can add value: On the robustness of the review but depth of discussion

 Smarter allocation of scarce resources – Decision modelling can demonstrate which services (such as reablement, falls prevention, or home adaptations) provide the best long-term outcomes for money spent, thereby reducing waste and extending independence.

 Fairness and equity – We emphasise the potential of newer approaches such as distributional cost-effectiveness analysis (DCEA) to highlight how benefits and costs are distributed across subgroups defined by age, disability, or income, helping policymakers prioritise both efficiency and equity.

 Improved comparability – We suggest a “minimum reporting set” for models (including base-case analysis, local budget impact, short/long horizon scenarios, and distributional results), which could make findings more actionable for decision-makers.

**On the conclusion**

 We have restructured the conclusion, so it does not end with a generic call for “more research needed.” Instead, we propose a pathway: standardisation of methods, explicit incorporation of social care outcomes, and inclusion of distributional analyses. This reframing emphasises that decision modelling can become a practical tool for guiding better social care decisions once these steps are taken.

**On the Plain English Summary**

 We appreciate your feedback and have substantially revised the Plain English Summary. The revised version now uses clearer language, includes concrete examples (reablement, falls prevention, home adaptations), and highlights the importance of fairness in resource allocation. We also note that we will seek input from people with lived experience and practitioners (PPI) to further improve accessibility and resonance.

 We are very grateful for your guidance, which has enabled us to make the paper stronger, more concrete, and more useful for both academic and policy audiences.

 Sincerely,

 Dacheng Huo

# Reviewer report: Reviewer response for version 2

**DOI:** 10.3310/nihropenres.15245.r36321

**Published:** 2025-08-27

**Authors:** Elizabeth Goodwin

**Affiliations:** 1 University of Exeter, Exeter, UK

No further comments

Is the review written in accessible language?

Yes

Are all factual statements correct and adequately supported by citations?

Partly

Are the conclusions drawn appropriate in the context of the current research literature?

Partly

Is the topic of the review discussed comprehensively in the context of the current literature?

Partly

Reviewer Expertise:

Health economics; economic evaluation of complex interventions; measurement of health and wellbeing outcomes. Prior to my academic career, I worked in planning, performance and project management for a local authority social services department.

I confirm that I have read this submission and believe that I have an appropriate level of expertise to confirm that it is of an acceptable scientific standard.

# Reviewer report: Reviewer response for version 1

**DOI:** 10.3310/nihropenres.14977.r34705

**Published:** 2025-02-26

**Authors:** Elizabeth Goodwin

**Affiliations:** 1 University of Exeter, Exeter, UK

Thank you for the opportunity to review this interesting paper on the use of decision modelling in the context of social care. This is a relevant and timely discussion, given the increasing interest in how economic evaluation methods can be applied to complex interventions that may sit partially or wholly outside the traditional “health” arena, and the extension of the remit of NICE to encompass adult and children’s social care. The paper explores the potential role of decision modelling in the evaluation of social care interventions before presenting a review that aims to illustrate the current state of play. This forms the basis for a discussion of key challenges and areas for future research and methodological development. This is a clear and well-written paper, which offers a useful contribution to the growing literature in this area, however I do have some suggestions for improvement.

 My primary concern is that there appears to be a mismatch between the content of the review and the rest of the paper; this is an issue that must be addressed to make the article scientifically sound. While the paper is framed throughout as focusing on social care interventions, this focus is not reflected in the studies included in the review. As a result of this, I have unfortunately concluded that the topic of the review has only partly been discussed in the context of the literature, and the conclusions drawn are only partly appropriate. The inclusion criteria for the review state that social care was defined as “practical support provided because of illness or disability”. In the UK, however, the working definition of social care is much more specific, and is typically limited to the types of interventions that may be commissioned or delivered by local authority social services departments. A number of the interventions included in this review, as described in Table 1 and in the original publications, do not meet this definition. For example, the six interventions reported by McDaid et al (2017) are *public health* interventions, the cognitive screening tests (Tong et al, 2017) are conducted by GPs in a *primary care* setting, and supported employment (Mavranezouli et al, 2014) is more likely to fall under the remit of the *Department for Work and Pensions*, eg via Job Centres. This is an important distinction, given that there are fundamental differences between social care and these other sectors in terms of their statutory responsibilities, primary intended outcomes, funding arrangements and evaluation guidelines. As such, it seems to me that this review covers the broader use of cost-effectiveness decision modelling in the evaluation of complex interventions that sit either partially or wholly outside the health sector (although under this definition it remains difficult to justify the inclusion of the cognitive screening tests), rather than its more specific use in the evaluation of social care interventions. I suggest either reframing the stated aims and focus of the paper along these lines, or limiting the included studies to those evaluating interventions that fall within the generally accepted definition and remit of social care.

 Other suggestions:

1. The methods employed to identify papers for inclusion in the review could be reported in greater detail. More information on search terms and strategies, and on the inclusion/exclusion criteria and how these were applied, would strengthen the paper. This could be provided as Supplementary Material.
2. The authors mention using the Drummond Checklist to assess the quality of the included studies, however the findings are not reported. A summary of this in the text would be informative, perhaps supported by an additional Supplementary Table.
3. There are several factual statements throughout the paper that are not supported by citations; please can these be added.

I have also had the benefit of reading the constructive and insightful review provided by our colleagues in Birmingham, and I support the points they have raised. Having said that, I’ve always been very fond of that George Box quotation, so for entirely non-scientific reasons I do hope that you keep it.

Is the review written in accessible language?

Yes

Are all factual statements correct and adequately supported by citations?

Partly

Are the conclusions drawn appropriate in the context of the current research literature?

Partly

Is the topic of the review discussed comprehensively in the context of the current literature?

Partly

Reviewer Expertise:

Health economics; economic evaluation of complex interventions; measurement of health and wellbeing outcomes. Prior to my academic career, I worked in planning, performance and project management for a local authority social services department.

I confirm that I have read this submission and believe that I have an appropriate level of expertise to confirm that it is of an acceptable scientific standard, however I have significant reservations, as outlined above.

# Response

**Published:** 2025-06-16

**Authors:** Dacheng Huo

**Affiliations:** University of York Centre for Health Economics, York, England, UK

Thank you for your constructive review and for acknowledging the relevance and contribution of our paper. Your comments have been very helpful in refining the clarity, scope, and positioning of our work. Below, we provide responses to each of your suggestions.

**Scope of the review and definition of social care**

 Thank you for raising this important point. We agree that conceptual clarity around what constitutes “social care” is essential, especially given the boundaries between health, public health, and social care in the UK context.

 In our review, we deliberately based the inclusion criteria on the scope of the ESSENCE Toolkit, a resource developed by the NIHR School for Social Care Research to support evidence-informed decision-making in adult social care. The Toolkit uses a broad but policy-relevant definition of social care derived from the National Audit Office (2018) framework, encompassing a range of services that provide practical support due to illness, disability, or age-related needs. This includes not only traditional local authority-provided care services, but also interventions related to housing, support, and community-based services that contribute to social care outcomes and resource use.

 We chose to adopt this framework for two reasons:

- First, the ESSENCE Toolkit is a curated, peer-reviewed source specifically intended to inform adult social care policy and commissioning decisions in England, making it directly relevant to our research aims.
- Second, the definition used in ESSENCE reflects the operational reality of social care delivery, where services are often delivered in partnership with or overlap with other sectors (e.g. health, employment, housing). These interventions, while not always under local authority remit, are frequently commissioned to improve outcomes for social care service users.

We acknowledge that our original manuscript did not sufficiently explain this rationale. In the revised version, we have added a detailed description of the ESSENCE Toolkit and its inclusion criteria, and clarified our decision to use it as the foundation for our review. We have also made it clear that our objective was not to define or restrict “social care” narrowly in legal or administrative terms, but rather to examine the current state of modelling practice as it applies to interventions intended to inform social care-related decision-making.

 We believe that this approach is methodologically appropriate and well aligned with the aims of our paper—to understand how decision modelling is being used (or underused) in the broader social care context, and to identify where future methodological work is most needed.

**Response to Other suggestions:**

1. **Methods: search process and inclusion criteria**

Thank you for suggesting more detail on our study selection process. In response, we have expanded the methods section to specify that the review focused on UK-based studies, which used decision modelling methods in the context of adult social care. We also clarified our use of the ESSENCE Toolkit as the main source of included studies, and added further explanation of the search scope and rationale. Additional details on search terms and selection criteria are now provided in the supplementary material to ensure transparency.

1. **Quality assessment reporting**

We appreciate your suggestion to report the findings of the quality assessment. We have revised the manuscript accordingly. We added the quality result based on Phillips checklist, which is better suited to evaluating decision modelling studies. A summary of the quality assessment is now included in the main text, and a detailed table has been added to the supplementary materials.

1. **Supporting citations**

Thank you for highlighting the need to reference key statements. We have reviewed the manuscript and added appropriate citations throughout. In particular, we now reference relevant literature to support claims about the strengths of decision modelling—such as its ability to extrapolate long-term outcomes and to integrate evidence from multiple sources through approaches like scenario analysis and meta-analysis.

 We are pleased to hear that you appreciated the inclusion of the George Box quotation. We have retained it in the revised version, as we agree it effectively captures the role of modelling in complex systems such as social care.

 We hope the revisions and clarifications made in response to your comments are satisfactory. Your feedback has been instrumental in improving the quality, clarity, and focus of our manuscript, and we are sincerely grateful for your contribution.

# Reviewer report: Reviewer response for version 1

**DOI:** 10.3310/nihropenres.14977.r34630

**Published:** 2025-02-08

**Authors:** Ian Litchfield, Melyda Melyda

**Affiliations:** 1 University of Birmingham, Birmingham, England, UK; 2 Department of Health Sciences, University of Birmingham (Ringgold ID: 152874), Birmingham, England, UK

Thank you for the opportunity to review this paper on decision modelling in social care. The topic is particularly relevant given the increasing recognition of the need for robust economic evaluations in social care settings. The paper is well-structured and provides an overview of the role of decision modelling in economic evaluation for social care interventions. It highlights the methodological challenges, the current state of decision modelling in the field, and areas for future development.

 While the paper is generally well-written and informative, there are a few areas where it could be strengthened.

1. Introduction: The opening quote, while insightful, does not clearly tie into the discussion that follows. Also, while the authors clearly state their objective—to *"….bridge existing gaps and enhance the robustness and applicability of decision modelling in social care settings*"—the introduction does not clearly define what the current gap is. It would also be helpful to clarify how this review differs from existing reviews of cost-effectiveness analyses of social care interventions (Bauer et al., 2021; Weatherly et al., 2017) and what specific contribution it makes beyond these prior studies.
2. Page 5: The statement, “ *While a healthcare QALY-based model may not be of primary interest in social care commissioning deliberations, a social care QALY-based model might do* (Stuttard et al., 2021),” would benefit from further elaboration. It is not entirely clear what the authors mean by a social care QALY-based model in this context and how it differs in application or relevance of the decision model. Would its use impact the decision model discussed in this section?  
3. Several claims regarding the benefits of decision modelling require additional referencing. For instance:

On Extrapolating over the longer term, and more specifically into the future section - " *Decision modelling allows this bridge between intervention today and outcomes over the longer-term to be made explicit, and for any necessary conditions for outcomes to be achieved, made clear*." (page 5)

 On Making comparisons across relevant interventions - "Decision modelling provides a framework to overcome these challenges through its ability to draw from multiple sources of evidence directly or use methodological approaches such as scenario analysis and meta-analysis." (page 6)

 Providing supporting references for these statements would strengthen the paper.

  

1. The criteria for selecting the reviewed studies are not entirely clear. It is not specified whether only UK studies were included, what years were considered, or what types of social care interventions were reviewed. The paper also does not specify where the review search was conducted (e.g., databases used, search terms applied).
2. The authors review decision models using the Drummond checklist, but I think the recommended approach for decision models is the Phillips checklist ( https://www.nice.org.uk/process/pmg20/resources/appendix-h-appraisal-checklists-evidence-tables-grade-and-economic-profiles-pdf-8779777885). I also wonder about the quality of those eight papers that were found.
3. **Page 7:** The statement, *“While reference was made to the complexity of social care decision-making in some of the studies (e.g. McDaid et al. (2017) and Public Health England (2018)), few attempts were made to reflect this complexity in the decision model, with the focus most often on costs falling on the public sector, specifically NHS and personal social services, and benefits to the intervention recipients,”* could benefit from further clarification. It is unclear what the authors mean by the **complexity of the model** in this context. How is complexity being defined, and what criteria are being used to assess it? Additionally, it would be helpful to elaborate on how the authors determine whether a model sufficiently captures the complexity of social care decision-making.
4. The authors frequently discuss social care outcomes but do not elaborate on the findings of the eight referenced papers. Unlike healthcare, where outcomes focus on clinical effectiveness (e.g., survival, QALYs), social care outcomes are broader and more complex, as shown in these studies. This might highlight the need for standardised metrics (e.g., ASCOT and ICECAP) to improve comparability and maybe consistency in decision models use in social care.
5. The review of existing decision models highlights the lack of a standardised approach, but the discussion does not fully explore how a more unified framework could be developed. Given the variability in modelling techniques and perspectives, some discussion on how consensus might be achieved—perhaps through a core set of recommended methods or guidelines—would be valuable.

Is the review written in accessible language?

Yes

Are all factual statements correct and adequately supported by citations?

Partly

Are the conclusions drawn appropriate in the context of the current research literature?

Partly

Is the topic of the review discussed comprehensively in the context of the current literature?

Partly

Reviewer Expertise:

Health Economics, Health Service Delivery

We confirm that we have read this submission and believe that we have an appropriate level of expertise to confirm that it is of an acceptable scientific standard, however we have significant reservations, as outlined above.

# Response

**Published:** 2025-06-16

**Authors:** Dacheng Huo

**Affiliations:** University of York Centre for Health Economics, York, England, UK

Thank you for your thoughtful and constructive review. We appreciate your recognition of the relevance and clarity of our manuscript. Your comments helped us refine the focus, improve the transparency of our methods, and strengthen the overall contribution of this review. Below we provide point-by-point responses.

 1. Introduction and objective clarity

 We have revised the introduction to more clearly define the gap addressed in this paper—specifically, the limited methodological development and inconsistent application of decision modelling in social care economic evaluation. The opening quote is now removed. We also clarified how this paper differs from existing reviews (e.g. Bauer et al., 2021; Weatherly et al., 2017) by focusing on the use of decision modelling itself, the challenges observed in practice, and potential directions for future development. The stated objective has been revised to better reflect this specific focus.

 2. Clarification of the “social care QALY-based model”

 We have revised this section to explain more clearly what is meant by a “social care QALY-based model.” Specifically, we now illustrate this using the example of the ASCOT tool, which captures domains such as independence, social connection, and control—outcomes that are highly relevant in social care contexts but not covered by conventional health-related quality of life measures. We also added a sentence explaining how decision modelling approaches can accommodate such outcome structures, provided that relevant states, transitions, and consequences can be appropriately conceptualized.

 3. Study selection criteria and use of the ESSENCE Toolkit

 Our review solely relied on the ESSENCE Toolkit. In the revised manuscript, we now provide a fuller explanation of why this source was appropriate. ESSENCE offers a comprehensive, policy-relevant collection of economic evaluations in adult social care and is based on a broad yet systematic definition developed by the National Audit Office. We clarified that the review focused on UK-based studies and added further details on databases and search terms.

 4. Quality assessment using the Phillips checklist

 Following your recommendation, we replaced the Drummond checklist with the Phillips checklist, which is more appropriate for assessing decision-analytic models. We updated the methods section accordingly and added a summary of the quality assessment results in both the main text and supplementary material.

 5. Clarifying the notion of “complexity” in models

 We did not apply a formal criteria for complexity, as this was not the central focus of the review and may not be appropriate across such a diverse set of studies. However, we revised the discussion to more clearly describe the types of complexity relevant to social care modelling—such as cross-sector costs and benefits, long-term impacts, and the involvement of multiple stakeholders. These issues were considered when commenting on how models reflected the complexity of real-world decision-making.

 6. Discussion on standardization and frameworks

 We agree that a more unified modelling framework would be valuable. However, proposing or outlining such a framework was beyond the scope of this review. Our aim was to map current practices and identify methodological gaps, rather than to offer prescriptive guidance. We now clarify this intention in the revised discussion and note that future methodological work could build on our findings to support greater standardization in the field.

 We hope that the revisions and clarifications we have made are in line with your suggestions. Your feedback has helped improve the clarity and focus of the manuscript, and we are sincerely grateful for your input.
